# A case report of successful treatment of advanced thymic squamous cell carcinoma with poor performance status using multimodal therapy

**DOI:** 10.3389/fonc.2024.1463772

**Published:** 2025-01-30

**Authors:** Xianhua Xu, Xiang Yan, Yang Qin, Dan Kou, Qi Huang, Qiongcheng Chen, Yejin Li

**Affiliations:** ^1^ Geriatric Medical Center, The General Hospital of Western Theater Command, Chengdu, China; ^2^ Department of General Medicine, The General Hospital of Western Theater Command, Chengdu, China

**Keywords:** thymic squamous cell carcinoma, pembrolizumab, carboplatin, albumin-bound paclitaxel, radiotherapy, poor PS

## Abstract

Thymic squamous cell carcinoma (TSCC) is a rare yet aggressive tumor associated with a dismal prognosis. The patient, a 76-year-old male, presented with symptoms of fatigue, bloody sputum and dyspnea. Computed tomography (CT) and positron emission tomography/computed tomography (PET/CT) scans demonstrated a significant mass in the chest, accompanied by metastasis to adjacent lymph nodes. Vascular ultrasound and CT angiography both indicated thrombosis in the jugular, innominate, and left subclavian veins. A CT-guided needle biopsy subsequently confirmed the diagnosis of TSCC. The patient had an Eastern Cooperative Oncology Group (ECOG) Performance Status (PS) score of 4 and exhibited high PD-L1 expression. The treatment plan entailed multiple administrations of low-dose carboplatin via intrapleural injection, infusions of albumin-bound paclitaxel (ABP), and pembrolizumab. Concurrent anticoagulation and nutritional therapy were also provided. A combination of carboplatin and ABP was administered for six cycles, followed by a single cycle of ABP alone. Subsequently, pembrolizumab was administered for ten cycles. After this, pembrolizumab was temporarily discontinued, and radiation therapy was administered to the primary tumor site and mediastinal lymph nodes. Five weeks later, pembrolizumab therapy was resumed. The patient’s PS has recovered to 0, and have survived for 23 months with a good quality of life. This experience suggests that combining pembrolizumab with low-dose chemotherapy drugs could be a beneficial treatment option for patients with advanced TSCC, poor PS, and high PD-L1 expression.

## Introduction

Thymic squamous cell carcinoma (TSCC) is a highly malignant subtype of thymic epithelial tumors (TETs) (1). Complete surgical resection remains the optimal treatment for patients without local invasion or distant metastasis (2). Chemotherapy, radiation therapy (RT), and immunotherapy can also help improve the quality of life for patients who are not eligible for surgery, although these treatments may exhibit limited efficacy and have adverse reactions. Additionally, chemotherapy is not recommended for patients with an Eastern Cooperative Oncology Group (ECOG) Performance Status (PS) of 3 or 4. Pembrolizumab, a programmed death-1 (PD-1) inhibitor, has been successfully used in treating various tumors (3). Compared to chemotherapy drugs that have significant side effects, pembrolizumab is well tolerated and effective. While the combination of chemotherapy and immunotherapy has achieved success in treating tumors, most patients undergoing these treatments have a good PS (4–6). However, there are few reports on the success of combining PD-1 inhibitors with dual-drug chemotherapy in patients with a PS of 4.

We present a case of advanced TSCC with multiple metastases and complications, initially assessed with a PS of 4. The patient was initially treated with a combination of chemotherapy and pembrolizumab, followed by RT. This case serves as an example of a treatment approach for TSCC patients with a poor PS, leading to continued tumor shrinkage and achieving partial remission (PR) that has lasted for over 23 months.

## Case present

A 76-year-old non-smoking Asian male, residing in a rural area at an altitude of 1200 meters, with a history of farming and working as a family doctor has a history of coughing and expectoration during cold seasons, which were not medically treated. There is no family history of cancer or genetic diseases, and he is mentally healthy with good social adaptability. In October 2022, he began to experience fatigue and occasionally noticed blood-streaked sputum. By December, he developed dyspnea. Due to severe motion sickness during travel, he did not seek medical examination or treatment. Three days prior to admission, his family doctor suspected a lung infection and prescribed intravenous levofloxacin 500 mg for two days, administered dexamethasone 5 mg for two days, and rehydration therapy, but there was no improvement.

**Figure 1:**
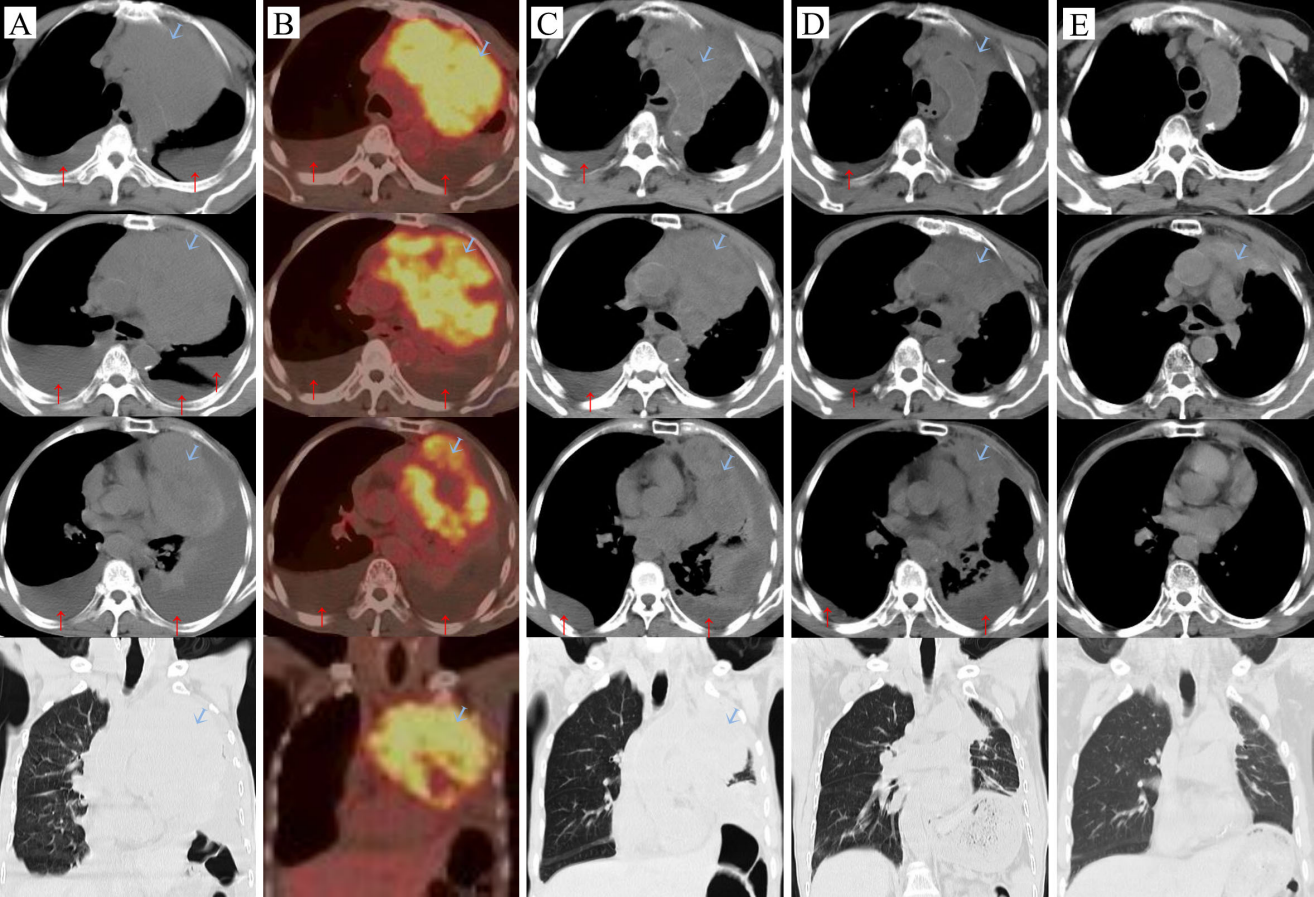
Carcinoma changes: 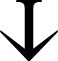 tumor, ↑pleural effusion. **(A)** Before the treatment (2023-01-09), **(B)** On the day of the first intravenous infusion of Paberizumab, PET/CT scan (2023-01-19), **(C)** After the first cycle of antitumor therapy (2023-02-09): carboplatin + ABP + Pembrolizumab, **(D)** Third cycle of carboplatin + ABP + Pembrolizumab (2023-03-27), **(E)** Fourteen months later (2024-03-26).

**Figure 2:**
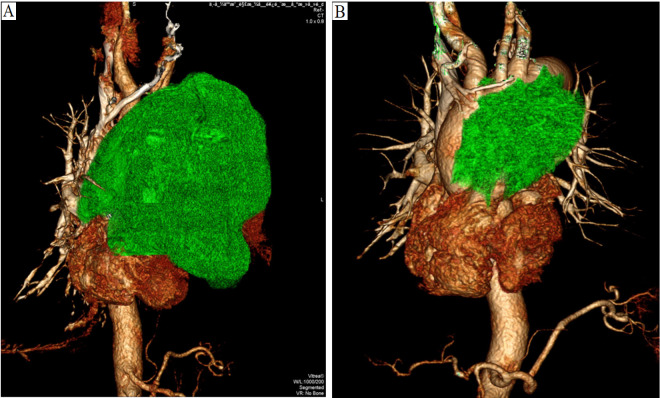
**(A)**. Enhanced CT angiography of the three-dimensional reconstruction reveals that the upper margin of the tumor extends beyond the aortic arch, while the lower margin reaches the inferior border of the heart, compressing the aortic arch, thoracic aorta, left pulmonary artery, and heart (2023-01-10). **(B)**. After a year of treatment, the tumor showed significant shrinkage (2024-02-23).

He was admitted in January 2023. A Physical examination revealed cachexia, a malnourished state, with distended left jugular veins, swelling of the left arm, absent breath sounds in the left lung, deviation of the trachea to the right, a heart rate of 130-145 beats per minute, a respiratory rate of 30-35 breaths per minute, and low blood oxygen saturation (88-91%) while on room air. Additionally, various blood parameters were abnormal (**Table 1**). Chest compute tomography (CT) scans showed a huge mass (13.9 cm×9.2 cm) in the anterior superior mediastinum (**Figures 1A**, **2A**), accompanied by elevated left diaphragm, bilateral pleural effusion, pericardial effusion, and enlarged mediastinal lymph nodes (**Figure 1A**). There was also bony destruction of the left 2nd to 4th ribs near the sternum and the left side of the sternum. A Positron emission tomography/Compute tomography (PET/CT) scan further revealed intense 18F-fluorodeoxyglucose uptake in the mass and lymph nodes (**Figure 1B**). Furthermore, vascular ultrasound and CT angiography demonstrated multiple venous thromboses. CT-guided biopsy and immunohistochemical analysis showed the following results: CD117(+), CD20(-), CD3(-), CD30(-), CD5(+), CD56 (focal+), CK19(+), CK5/6(+), CK7(-), CK8/18(+), CR (focal+), PAX-5(-), KI67(+15%), OCT3/4(-), P63(+), PAX-8(-), Syn(-), TTF-1(-), TDT(-), and WT-1. Based on cell morphology, along with positive CD117(+) and CD5(+) and negative TTF-1(-), lung squamous cell carcinoma was ruled out, leading to a diagnosis of TSCC (7, 8). According to the Masaoka-Koga staging system, the patient was diagnosed with stage IVb TSCC (T4N2M1b) and a performance status (PS) of 4. Concurrent diagnoses included lung infection, respiratory failure, cachexia, anemia, hypoproteinemia, deep venous thrombosis, and chronic bronchitis. Given the patient’s large tumor, bilateral malignant pleural effusion, and dyspnea, his initial prognosis was poor, with an expected survival of less than two months.

**Table 1:** Blood test results at admission.

Test	Results	Normal value
Leukocyte	12820cell/ul	3500-9500cell/ul
Hemoglobin	11.2g/dl	13.0-17.5g/dl
Lymphocyte	64cell/ul	110-320cell/ul
Platelet	542000cell/ul	125000-35000
D Dimer	8.08ng/L	0-0.55ng/L
Albumin	35g/dL	40.0-55.0g/dL
C-reactive protein	96.01ng/L	0-3mg/dL
Procalcitonin	0.2ng/ml	0-0.05ng/ml
PD-L1 expression	≥50%	–
Ca125	175.5U/ml	0-35U/ml
(CYFRA21-1)	3.88ng/ml	0-0.33ng/ml

**Figure 3:**
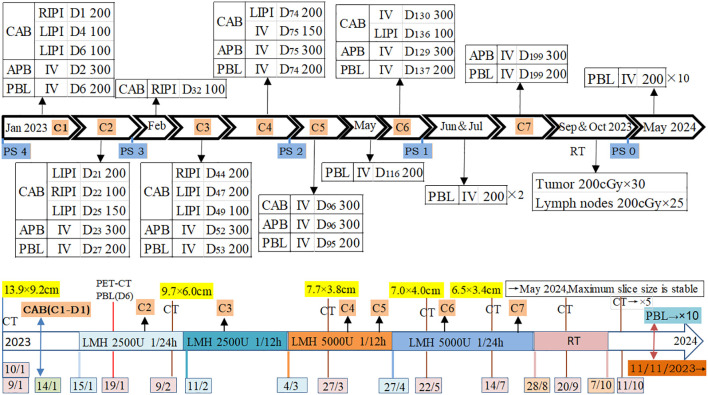
Time and method of drug use, as well as tumor changes. C: Cycle; D: Day; CAB: Carboplatin (mg); ABP: Albumin-bound paclitaxel (mg); PBL: Pembrolizumab (mg); RIPI: Right intrapleural injection; LIPI: Left intrapleural injection; IV: Intravenous drip; RT: Radiation therapy; LMH: Low molecular weight heparin; PS: Performance Status; CT: Compute tomography; PET/CT: Positron emission tomography/Compute tomography; CAB(C1-D1): Carboplatin (administered on the first day of the first cycle).

After admission, the patient received treatments including oxygen therapy, intravenous and oral nutritional support, antibiotics, and pleural catheter placement to drain blood from the pleural effusion. Due to the patient’s frail condition and the high risks associated with high-dose intensive chemotherapy, and with full understanding and written consent from the patient and his family, on January 14th, 200 mg of carboplatin was injected into his right pleural cavity (RPC). The patient experienced no significant adverse reactions. The next day, 300 mg of albumin-bound paclitaxel (ABP) was administered intravenously. The patient’s fatigue worsened for a day but subsequently improved. On the fourth and sixth days, 100 mg of carboplatin was injected into his left pleural cavity (LPC) at different intervals. Test results on the sixth day showed that the expression level of programmed death-ligand 1 (PD-L1) was ≥50% (22C3), so 200 mg of pembrolizumab was administered intravenously without adverse reactions. Fifteen days after the administration, the color of the pleural effusion began to lighten and gradually decreased. The patient’s dyspnea symptoms improved, confirmed the effectiveness and safety of this treatment strategy. This treatment plan was continued, with carboplatin doses ranging from 100 mg to 200 mg administered into the pleural cavity under ultrasound guidance, based on the volume of pleural effusion, the patient’s physical condition, and their response to the medication. During the fourth cycle, 200 mg of carboplatin was injected into the pleural cavity (LPC), along with 150 mg of intravenous carboplatin. In the fifth and sixth cycles, 300 mg of carboplatin was administered intravenously on the same day as ABP, and an additional 100 mg was injected into the LPC during the sixth cycle (**Figure 3**). Specifically, based on the patient’s PS and blood test results, the interval between administrations of ABP and pembrolizumab was set at 21 days or more.

During the treatment process, as the tumor shrank and the pleural effusion decreased (**Figures 1C, D**) the patient’s dyspnea gradually resolved, and his physical strength improved while his weight slowly increased, PS has gradually recovered (**Figure 3**). At the end of the seventh course, the pleural effusion had completely disappeared. The main side effects of chemotherapy were bone marrow suppression and gastrointestinal reactions. During the third and fourth courses, when the pleural effusion was reduced, he experienced a fever for one day after the intrapleural injection (IPI) of carboplatin, which resolved on its own. However, after the intravenous infusion, he had a fever for 3 to 4 days and required symptomatic treatment. Nevertheless, due to the effectiveness of carboplatin in reducing pleural effusion, it was not replaced. Anticoagulation therapy was also administered concurrently. Based on changes in the patient’s hemoptysis and bloody pleural effusion, the dose of low-molecular-weight heparin was increased from 2,500 U once daily to 5,000 U every 12 hours and then gradually reduced until the end of radiotherapy (**Figure 3**). The patient’s left jugular vein returned to normal, and the swelling in his left arm disappeared. Ultrasound showed that his left jugular vein and left subclavian vein were recanalized, although there was thrombosis in the left innominate vein.

After completing chemotherapy and ten cycles of pembrolizumab treatment, the patient discontinued pembrolizumab and underwent radiotherapy for the mediastinal lesion (200 cGy×30) and lymph nodes (200 cGy×25). Five weeks later, the patient experienced no discomfort, and all blood tests were normal. Therefore, pembrolizumab maintenance therapy was resumed (**Figure 3**). In both the February 2024 enhanced CT reconstruction and the March 2024 CT scan, it showed that the largest cross-sectional dimension of the tumor was 6.3 cm × 3.0 cm, and there was no recurrence of pleural effusion (**Figures 1E**, **2B**). However, CT scans of the lungs revealed mild interstitial changes in the left upper lobe near the tumor. We extended the interval between pembrolizumab treatments. As of the revision of this article in December 2024, imaging examinations show no new tumor metastasis or pleural effusion, and the blood test results are normal. He has maintained a good quality of life for over 23 months, with a PS of 0.

## Discussion

This case involves a patient with a large chest tumor accompanied by severe physical symptoms, radiographically diagnosed with sternal and rib metastases, as well as bilateral massive hemothorax indicating pleural metastasis. After approximately six months of combined immunotherapy and chemotherapy, the tumor markedly shrank, and the patient’s PS improved to 1. This positive outcome was likely attributed to the administration of pembrolizumab in combination with chemotherapy, based on the high PD-L1 expression in the tumor. Notably, the tumor’s longest diameter decreased by approximately 4 cm during the first cycle of treatment, with pembrolizumab potentially played a pivotal role in tumor reduction. Previous studies have also demonstrated that patients with advanced thymic carcinoma who are chemotherapy-refractory but have high PD-L1 expression respond better to pembrolizumab (1, 9). Intrathoracic injection of carboplatin allowed the pleural lesions to be exposed to higher drug concentrations (10), which may have played a major role in reducing or eliminating pleural effusion. In this case, the combination of immunotherapy and chemotherapy synergistically treated the patient’s large tumor, aligning with previous reports on combination therapy for squamous cell carcinoma (4), also in accordance with the reported treatments for TSCC (11). The rapid tumor reduction and resolution of hemothorax alleviated the patient’s dyspnea and facilitated physical recovery, laying the foundation for subsequent treatment.

For large tumors, neither immunotherapy nor chemotherapy serves as a radical treatment strategy. Therefore, RT is crucial for preventing recurrence. RT is a commonly used treatment for TETs, albeit with a potential risk of inducing radiation pneumonitis (RP) (12). A meta-analysis has shown that the use of PD-1 inhibitors increases the risk of pneumonia at all exposure levels (13). Additionally, platinum-based drugs and paclitaxel may further elevate this risk (14). Compared to concurrent therapy, sequential RT and chemotherapy may carry a higher risk of RP (15). Despite these potential risks, radiotherapy maximizes the reduction of tumor recurrence in cases of large tumors. However, chronic radiation pneumonitis may develop within 12 weeks to 6 months (16), and the continued use of pembrolizumab necessitates ongoing monitoring for the onset of pneumonitis.

It should be noted that this observation is only a case report for TSCC. Limitations of this case’s treatment process include the absence of targeted drug genotyping to validate efficacy and the absence of a follow-up PET/CT scan and histological evaluation following tumor reduction.

The patient was amazed by the treatment outcome, felt confident about the future, and was reluctant to undergo further histological follow-up.

## Conclusion

For patients with advanced TSCC and poor performance status (PS), the combination of PD-1 inhibitors and low-dose chemotherapy may represent a beneficial treatment option, particularly for those with high PD-L1 expression.

## Data Availability

The raw data supporting the conclusions of this article will be made available by the authors, without undue reservation.
